# Connectome-Guided Personalization of Optimal TDCS Intervention Selection in Alzheimer's Disease: A Modeling Study

**DOI:** 10.1523/ENEURO.0407-25.2026

**Published:** 2026-08-11

**Authors:** Janne J. Luppi, Annel P. Koomen, Cornelis J. Stam, Philip Scheltens, Willem de Haan

**Affiliations:** ^1^Alzheimer Center Amsterdam, Department of Neurology, Amsterdam UMC, Amsterdam 1081 HZ, The Netherlands; ^2^Amsterdam Neuroscience, Vrije Universiteit Amsterdam, Amsterdam 1081 HZ, The Netherlands; ^3^Department of Clinical Neurophysiology and MEG, Amsterdam UMC, Amsterdam 1081 HZ, The Netherlands; ^4^EQT Life Sciences Dementia Fund, Amsterdam 1071 DV, The Netherlands

**Keywords:** Alzheimer’s disease, neural mass model, personalization, transcranial direct current stimulation

## Abstract

Transcranial direct current stimulation (tDCS) could reduce the neurophysiological effects in Alzheimer's disease (AD), but progress is hampered by variable outcomes across studies, likely related to both methodological and individual differences. We recently described a virtual brain network simulation method for optimizing tDCS interventions and now propose a method for further personalizing this approach. We now personalized the model for six female and four male biomarker-confirmed AD patients based on their brain structure and functional connectivity by using individual structural magnetic resonance imaging data and amplitude envelope correlation-based connectivity matrices extracted from magnetoencephalography (MEG) scans, respectively. We then assessed a set of previously established stimulation strategies based on their ability to improve relevant neurophysiological outcome parameters in each personalized model while undergoing AD damage. Personalized tDCS strategies were able to delay neurophysiological deterioration, but while the general model favored posterior anodal stimulation targeting the precuneus region, the personalized models favored frontal anodal stimulation targeting the dorsolateral prefrontal cortex region in 90% of the cases. This may be explained by higher connectivity levels of frontal regions in the personalized connectivity matrices, as anodal stimulation of highly connected regions produced more beneficial effects. In this methodological study, we propose several ways to improve personalized computational tDCS stimulation prediction modeling. We conclude that connectome-guided personalization of tDCS effects lead to different strategies with potentially better intervention outcomes. For external validation of this model-guided tDCS approach, model predictions are being tested in an ongoing clinical tDCS–MEG trial in AD patients.

## Significance Statement

Our aim is to improve the efficacy of transcranial direct current stimulation (tDCS) in Alzheimer's disease through the use of a personalized modeling approach. This is beneficial since results of tDCS remain variable across groups, and therefore a systematic approach to choosing the stimulation parameters such as placement of electrodes is required. Modeling of tDCS allows us to do this while assessing multiple possible approaches without necessitating a considerable burden on patients across multiple trials. Furthermore, personalizing the model can enable more accurate selection of target regions for stimulation, for example, by locating highly connected brain regions without too much damage in each patient, thus increasing the potential for recovery.

## Introduction

Noninvasive brain stimulation in Alzheimer's disease (AD) has shown promise as an alternative treatment to pharmacological interventions (Reinhart et al., 2017; Chang et al., 2018; Reinhart and Nguyen, 2019; Benussi et al., 2021, 2022; Yang et al., 2021; Chan et al., 2022; Koch et al., 2022). While recent amyloid-targeting drug treatments have improved (van Dyck et al., 2022), they are expensive, intensive, and not without side effects, justifying alternative approaches. Transcranial direct current stimulation (tDCS) is a noninvasive brain stimulation method in which a low current is passed from a positive anode to the negative cathode in order to stimulate or inhibit the neuronal excitability of targeted regions (Nitsche and Paulus, 2000, 2001; Nitsche et al., 2003). The primary effects of tDCS are understood to be on pyramidal neurons aligned perpendicular to the cortical surface, aligned with the current. Under the anode, the soma and axon of these cells become depolarized, therefore facilitating action potential generation, while an inhibitory effect occurs under the cathode (Bikson et al., 2004; Lewis et al., 2025). By regulating disturbed levels of neuronal activity, tDCS could potentially be used to alleviate, for example, excitotoxicity, which is one of the primarily amyloid-related neurophysiological damaging effects in AD (Bero et al., 2011; Harris et al., 2020). Moreover, shifting of neuronal activity toward normal ranges can potentially lead to an improvement in neuronal network integrity, delaying the decay of cognitive processing.

The clinical appeal of this safe and patient-friendly approach is intervening directly at the level of brain function, which has a stronger relation with cognitive status than the underlying pathology (Jeong, 2004; Sperling et al., 2010; Marceglia et al., 2016; Palop and Mucke, 2016; Antal et al., 2017; Murugaraja et al., 2017). Indirect support for effectiveness of therapy that targets neuronal dynamics comes from other neurological conditions (e.g., antiepileptic drugs, deep brain stimulation in Parkinson's disease) and is in line with the currently preferred AD medication restoring cholinergic deficiency, which primarily acts on neuronal signal transmission (Birks and Grimley Evans, 2015; McShane et al., 2019). Furthermore, since disrupted neuronal activity can reinforce pathological protein depositions, targeting this disrupted activity with restorative stimulation might be able to reduce the pathological burden of AD (Bero et al., 2011; Harris et al., 2020). Noninvasive brain stimulation can also relevantly promote neuroplasticity, which is not targeted with current pharmacological approaches (Vestring et al., 2024). Finally, while tDCS current flow does spread more widely on the cortex compared with more focal stimulation techniques, it is capable of regional targeting, which enables more precision and diversity in interventions when compared with systemically acting pharmacological treatments.

Despite these promising qualities, a great challenge in the field of tDCS is the variability in results. While several studies have found improvements in response to tDCS in both neuropsychological and clinical cognitive measures, other studies report little to no effect (Cotelli et al., 2014; Bystad et al., 2016; Hill et al., 2016; Liu et al., 2020; Antonenko et al., 2021; Antal et al., 2022; Menardi et al., 2022). This variability is likely partly caused by differing experimental setups, especially in terms of electrode placement. Often, electrode placement is motivated by directly targeting relevant cortical regions based on anatomy or pathology (Sperling et al., 2009; Gangemi et al., 2021; Koch et al., 2022). This approach omits more distant, nonlinear network effects, which could dampen or even reverse the intended effects. Also, different stimulation regimes with regard to intensity, polarity, and duration can lead to different results (Nitsche and Paulus, 2000). This fuels the search for optimal procedures in a systematic way (Menardi et al., 2022).

There is an increasing need to personalize tDCS intervention to best suit individual anatomy, network connectivity and pathology (Antal et al., 2017; Antonenko et al., 2021). For example, certain regions may have suffered too much atrophy in some patients to be good targets for stimulation, while other highly connected regions may predict excellent stimulation targets with network-wide intervention effects. With the increasing recognition of AD variants (posterior cortical atrophy, dysexecutive, frontal, corticobasal) comes also the notion that these might be treated differently (Ossenkoppele et al., 2015; Di Stefano et al., 2016; Groot et al., 2020).

Computational brain network modeling is one option for systematically addressing the issue of variability in brain stimulation research without burdening patients across countless clinical trials (Miniussi et al., 2013; de Haan et al., 2017; Bassett et al., 2018; Luppi et al., 2023). Neurophysiological network models can be used to run simulations in order to investigate the theoretical effectiveness of a large number of stimulation setups, including unintuitive setups which could still prove beneficial due to unexpected network interactions (Sporns, 2014; Stam, 2014; Bassett and Sporns, 2017; Bassett et al., 2018; Luppi et al., 2023). Combining techniques such as current flow modeling (CFM) and computational network models can be used to predict the downstream effects of the stimulation throughout networks (Thielscher et al., 2015; de Haan et al., 2017).

The aim of the current study is to build on our previous modeling work (Luppi et al., 2023) and add personalization by implementing individual anatomy and connectivity into a neural mass model of the AD brain. We determined which strategy from a previously established set of electrode montages performed best in each individual, and whether this selection differed between individuals and from our previous results in a model without personalization. Since notable differences between individuals in structural and functional connectivity have been described, both in health and disease conditions, this might have consequences for therapy (Bajo et al., 2010; Engels et al., 2015).

Our outcome parameters were kept the same as in our prior modeling study (Luppi et al., 2023), where they were chosen to assess oscillatory slowing (total power, relative power in the lower and upper alpha bands and posterior dominant peak frequency) and loss of functional connectivity [corrected amplitude envelope correlation (AEC) and phase lag index]. Oscillatory slowing, observed in a shift from higher frequencies (alpha) to lower (delta, theta) especially in occipital regions, is well documented in AD (Jeong, 2004; Babiloni et al., 2013; Engels et al., 2017; Giustiniani et al., 2023) and has been associated with disease severity and pathogenic protein load (Rudolf et al., 2022; Scheijbeler et al., 2023). As the model we use features primarily alpha frequency activity, we have focused on this frequency band, but we also included total spectral power and posterior dominant frequency to assess overall spectral changes. A loss of functional connectivity has also been reported in AD cohorts, including in the measures of our choice, and its extent has been associated with disease severity (Engels et al., 2017; Babiloni et al., 2020; Briels et al., 2020; Schoonhoven et al., 2022). As such, based on the current literature, we argue that being able to elicit a shift in these disrupted outcome measures toward healthy values would also indicate potential for clinical improvement in cognition.

We hypothesized that implementing individual anatomy and connectivity into our systematic modeling approach for optimal tDCS electrode placement in AD would result in different electrode placements performing better in certain individuals than others. We expected stimulation aimed at highly connected (hub) regions to perform better. External validation for the model-guided tDCS predictions will be explored in a recently completed clinical trial in which AD patients have received both general and personalized tDCS interventions with simultaneous magnetoencephalography (MEG).

## Materials and Methods

**Figure 1.**
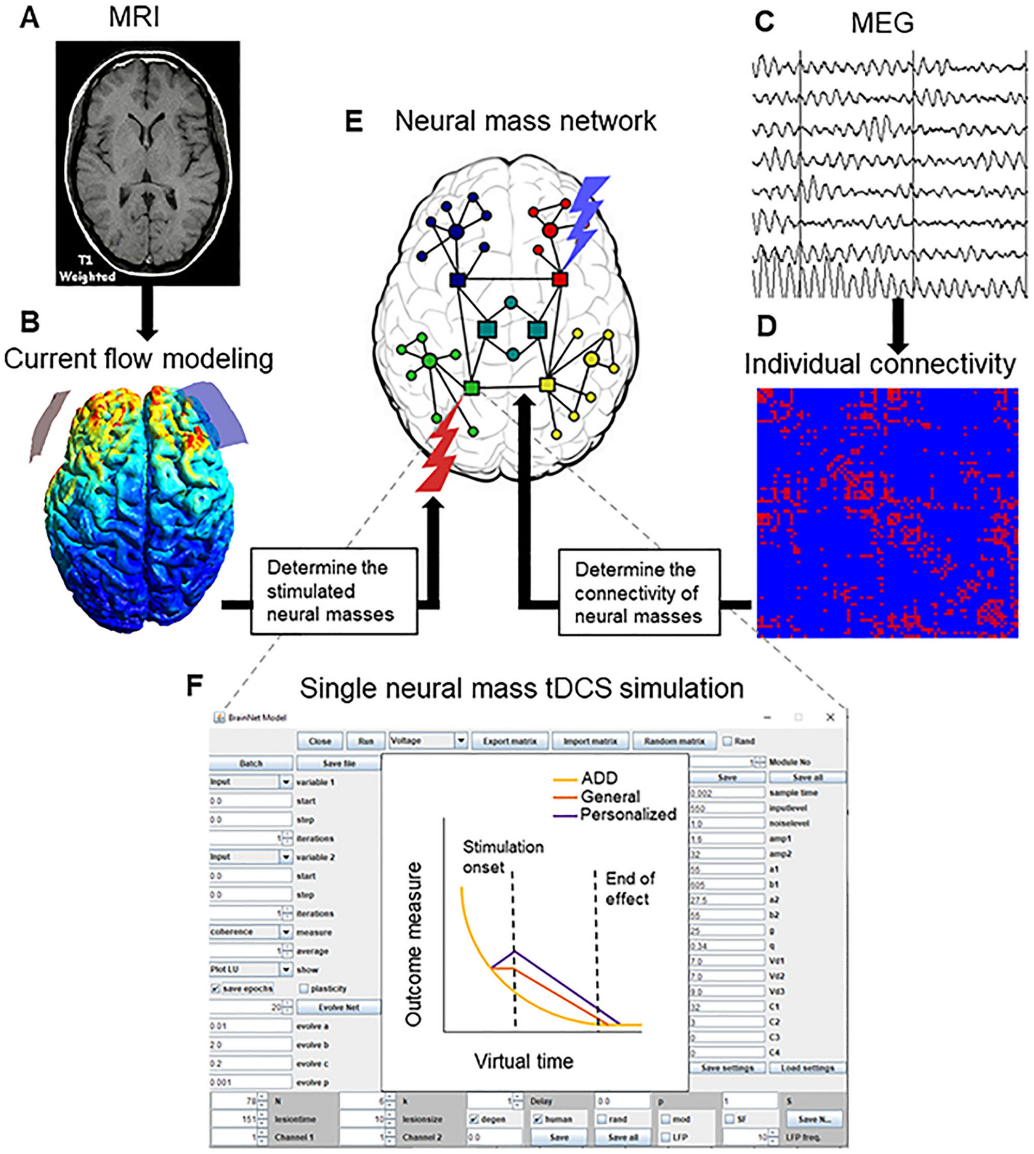
Model personalization outline. ***A***, Individual T1-weighted images were used to generate head meshes for CFM. ***B***, CFM was used to determine which AAL atlas regions and the corresponding neural masses were stimulated in each stimulation setup. ***C***, Individual MEG scans were used to calculate the AEC connectivity. ***D***, Individual connectivity matrices were imported into the model. ***E***, A schematic of the neural mass model network shows interconnected neural masses. Targeted neural masses and the connectome of the network are based on steps ***B*** and ***D***. ***F***, Simulations are evolved over virtual time using the activity-dependent damage algorithm (ADD) without intervention and then again combined with all six stimulation setups. The best-performing strategy in the personalized model is compared with the strategy that performed best in the general model, which is now implemented in the personalized model.

For this methodological study, we selected a subgroup that was representative of our clinical trial cohort. The personalization procedure of our computational model is based on the integration of individual MEG and magnetic resonance imaging (MRI) data. Please refer to Figure 1 for an outline of the procedure.

### Participants

MRI data of 10 subjects with a biomarker-confirmed early–stage AD dementia diagnosis were included for this methodological computational modeling study. Six of the participants were female, and the group had a mean age of 60.9 (±5.9) years and an average MMSE of 23.5 (±3.5). All participants were recruited from the Amsterdam Dementia Cohort in the period of 2022–2025 (van der Flier and Scheltens, 2018). All participants had been assessed according to a standard clinical protocol, which involved history taking, physical and neurological examination, an interview with a spouse or close family member, blood tests, 3 T MRI of the brain according to a standard protocol, routine MEG, a neuropsychological assessment battery, and a lumbar puncture or an amyloid-β positron emission tomography (PET). Subjects with a clinical diagnosis of dementia due to AD according to National Institute on Aging–Alzheimer's Association, and with positive amyloid-β biomarkers (cerebrospinal fluid ptau/ amyloid-β ratio >0.020 and/or abnormal amyloid-β PET) were recruited. The mean time between diagnosis and inclusion was 240 d (max 480 d; SD, ±160 d). The local Research Ethics Committee approved the study, and all participants gave written informed consent.

### MEG data acquisition and preprocessing

MEG recordings were obtained as part of the subjects' clinical evaluation during the standard diagnostic process at the memory clinic. MEG recordings were performed in a magnetically shielded room (VacuumSchmelze) using a 306-channel whole-head Triux Neo system (MEGIN Oy). The recording protocol consisted of a 10 min block of eyes-closed, resting-state condition. During the scan, patients were instructed to close their eyes, stay awake, and reduce eye movements. The recordings were sampled at 1,000 Hz, with an online anti-aliasing filter (330 Hz) and high-pass filter (0.1 Hz). The temporal extension of signal space separation (Taulu and Simola, 2006), as implemented in the MaxFilter software (Elekta Neuromag Oy, version 2.2.12), was applied with a sliding window of 10 s and correlation limit of 0.9. After visual inspection, channels containing excessive artifacts were manually removed. The head position relative to the MEG sensors was recorded continuously using the signals from five head-localization coils. The head-localization coil positions and the outline of the participant's scalp (∼2,500 points) were digitized (FASTRAK, Polhemus). This scalp surface was used for coregistration with individual T1-weighted MRI scans.

### MEG source reconstruction

In order to obtain source-localized activity for all regions, we applied an atlas-based beamforming approach (Hillebrand et al., 2005). Sensor signals were projected to an anatomical framework such that source-reconstructed neuronal activity for 78 cortical regions of interest (ROIs), identified by means of automated anatomical labeling (AAL), was obtained (Tzourio-Mazoyer et al., 2002). To obtain a single time series for an ROI, we used each ROI’s centroid as representative for that ROI. For the computation of the beamformer weights, we used the sphere that best fitted the scalp surface as a volume conductor model and an equivalent current dipole as source model. The orientation of the dipole was chosen to maximize the beamformer output. Once the broadband (0.5–48 Hz) normalized beamformer weights for the selected voxel were computed, then the broadband (0.5–48 Hz) time series for this voxel, i.e., a virtual electrode, was reconstructed.

### Data selection and processing

The source-reconstructed time series were converted to ASCII files for further processing. For each subject, 10 visually selected artifact-free epochs without downsampling (sample frequency 1,000 Hz, 4,096 samples per epoch of 4.096 s) of the eyes-closed, resting-state recording were analyzed. All quantitative spectral analyses were performed with in-house developed software (BrainWave version 0.9.164.16). Broadband power and relative power were calculated using the fast Fourier transform with the following bands: broadband (0.5–45 Hz), delta (0.5–4 Hz), theta (4–8 Hz), lower alpha (8–10 Hz), upper alpha (10–13 Hz), beta (13–30 Hz), and gamma (30–48 Hz). Peak frequency of the posterior dominant rhythm was calculated in the 4–13 Hz range in six posterior AAL regions in each hemisphere (superior, middle, and inferior occipital gyri, calcarine region, cuneus, and lingual gyrus) and then averaged to obtain a single value per epoch.

### Neural mass model

The model used is comprised of interconnected neural masses, which represent interconnected populations of excitatory and inhibitory neurons. This neural mass model is an adjusted version based on the original by Lopes da Silva et al., which has been optimized for reproducing the human alpha rhythm (8–13 Hz; Lopes da Silva et al., 1974, 1976; Zetterberg et al., 1978; Stam et al., 1999). The model has been used for several network studies on AD, due to the relevance of the alpha rhythm in characterizing the oscillatory slowing in AD (de Haan et al., 2012, 2017; Luppi et al., 2023). The neural masses generate an oscillatory output comparable to MEG, relating neuronal circuit characteristics to cortical activity. For more detailed model information, please refer to previous studies (de Haan et al., 2012, 2017; Luppi et al., 2023).

The 78 neural masses of the model correspond to the cortical regions of the AAL atlas and are coupled according to human brain topology (Tzourio-Mazoyer et al., 2002). Prior to personalization, this topology is based on diffusion tensor imaging (DTI) results by Gong et al., describing average structural connectivity of the cortex of 80 healthy adults (Gong et al., 2009). This was the general connectivity matrix used in our previous study without personalization (Luppi et al., 2023). All connections between neural masses were excitatory and reciprocal, meaning that without external influence, the differences in activity arose from the pattern of connections.

### The activity-dependent degeneration (ADD) algorithm

To simulate damage in the network caused by AD, a previously described algorithm of ADD was used in the model (de Haan et al., 2012, 2017). The ADD algorithm damages the network by lowering the coupling strength within and between neural masses as a function of recent spike density of the main excitatory population. This means that the higher the activity level in a neural mass, the more the structural connectivity of that neural mass is damaged over virtual time in the model. Since the current model has no plasticity features, i.e. no option to strengthen or regain connections, the ADD algorithm inevitably leads to network breakdown.

### CFM

CFM was carried out in the free SimNIBS software package (Thielscher et al., 2015). CFM uses MRI data to delineate tissue types within a head and assigns them corresponding conductance properties in order to predict how current spreads within the cortex for a given tDCS electrode montage. The current spread results were used at a later stage of the pipeline to determine which parts of the neural mass model would be excited or inhibited in each investigated virtual tDCS montage.

**Figure 2.**
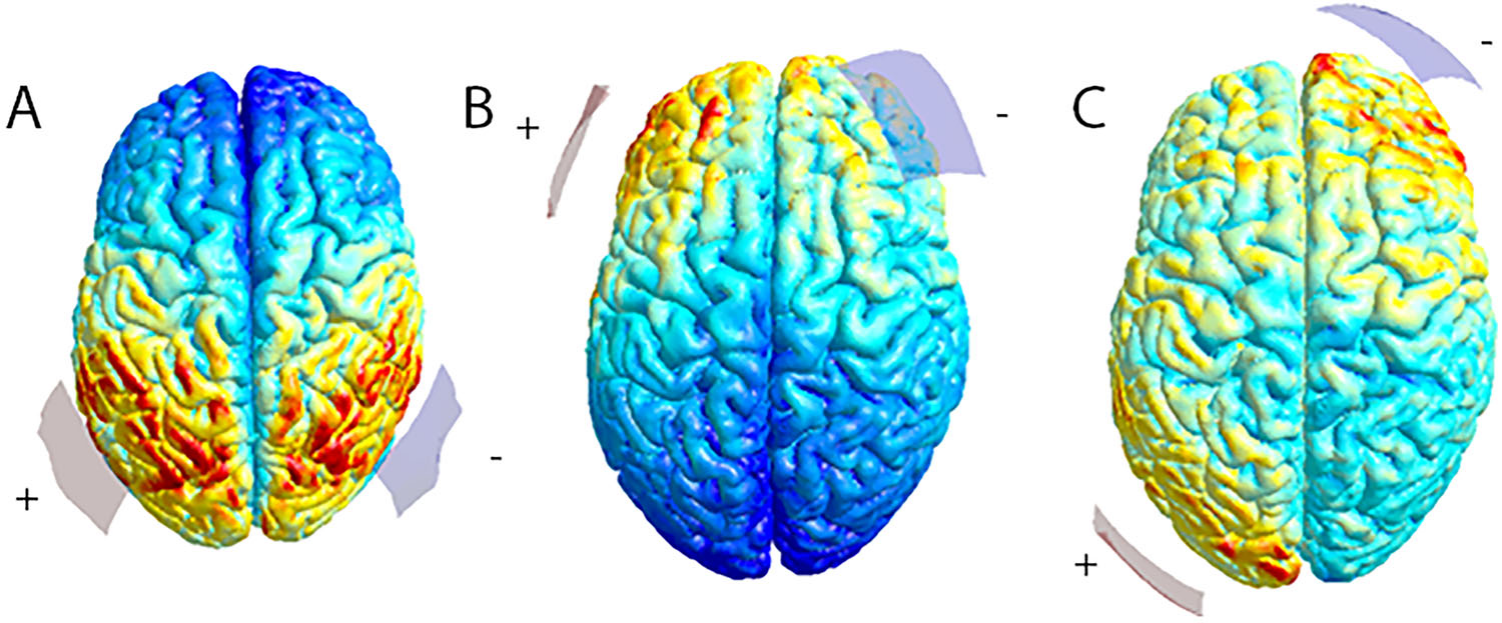
Electrode montages. The three figures show each unique electrode position used, with the red electrode being the anode and the blue electrode being the cathode. The associated plot of the cortex displays the associated CFM done in SimNIBS (Thielscher et al., 2015). ***A***, The bilateral posterior montage, with anode at P5 and cathode at P6. Bilateral precuneal region is a large, integrative multimodal association network hub and close to the cortical posterior dominant rhythm, which gradually slows down in AD (Huang et al., 2000; Babiloni et al., 2004; Jeong, 2004). ***B***, The frontotemporal montage, with the anode at F7 and cathode at F4. The left dlPFC and left inferior frontal cortex have stood out in studies that improved working memory (Wischnewski et al., 2021). ***C***, The parietofrontal montage, with the anode at PO7 and cathode at AF4. This strategy targets parieto-occipital regions, including the precuneus (Engels et al., 2015; Koch et al., 2022). Studies targeting temporoparietal and parieto-occipital areas have shown clinical improvement (Ferrucci et al., 2008; Marceglia et al., 2016; Khedr et al., 2019). For each listed montage, a hemispherically mirrored version was also used, e.g., in addition to an anode at F7 and a cathode at F4, a reversed position with an anode at F8 and a cathode at F3 was used.

In order to create personalized head models, the *cure* pipeline provided with the software was used to transform a T1 MRI image of each subject into a head mesh used in the software. The software then used this head mesh to calculate the current spread in the cortex based on the provided electrode positions. The six electrode positions chosen for the simulations are described in Figure 2, in which three unique positions and their hemispherically mirrored versions are included (Luppi et al., 2023). These positions were chosen due to them performing the best in the previous study as well as a preliminary analysis which showed this to be the case also in the personalized model. The montage of PO8 anode and AF3 cathode performed best in the general model and will therefore be referred to as the general strategy, although in this study it is applied in the personalized model. Simulations were carried out using 5 × 5 cm gel electrodes.

To interpret the results, the head plots from the CFM pipeline were visually delineated into the regions of the AAL atlas, which correspond to the neural masses of the model. A region was affected by the stimulation if electric field strength exceeded 0.6 of the maximum field strength in at least 50% of the region. Furthermore, each affected region was determined to be anodally stimulated if it was included in the electric field closest to the anode or cathodally stimulated if it was included in the electric field closest to the cathode. All implementations of the CFM results into the model were carried out by the same rater.

### Individual connectivity matrices

As a measure of individual structural connectivity, the uncorrected AEC was calculated in the alpha range (8–13 Hz) based on the MEG scans of each subject (Hipp et al., 2012; Schoonhoven et al., 2022). Because this AEC has no correction for volume conduction, it retains much of the structural connectivity information, as both are heavily influenced by distance between regions (Millán et al., 2022, 2024). It was therefore chosen as a proxy for the structural connectivity, in order for the personalized networks to be comparable to the general DTI structural network (the Gong matrix). Furthermore, since it is based on brain dynamics instead of structure, it may be more closely related to the regional activity and functional connectivity targets we are trying to improve.

The AEC was obtained for a 78 × 78 connectivity matrix of corresponding to the cortical regions of the AAL atlas. The AEC connectivity matrix was then imported into the model prior to each simulation as the personalized matrix replacing the general DTI-based average human 78 × 78 connectivity matrix (AAL). In order to match the average connectivity of the original binarized DTI matrix with 10% sparsity while retaining the new non-binarized information, the 15% strongest connections were kept in the generated AEC connectivity matrix. A personalized matrix averaged across the personalized matrices of all subjects was also compared with the general matrix in order to assess overlap and differences their structures.

Finally, we created an averaged AEC matrix. For this, all 10 individual AEC matrices were averaged to generate a new matrix that was then used to model strategy success in a new “general” model but now using an AEC matrix instead of a DTI matrix as was done in our previous study. This was done as a control to assess to what degree differences between the personalized models and the previous general model were caused by individual differences or by methodological differences between the AEC and DTI matrices. All simulations were performed the same on this model as for the personalized models.

### Virtual tDCS simulations

After importing the personalized connectivity matrices into the model, simulations were carried out in eight conditions for each subject. In the healthy control (HC) condition, the model was run for 30 virtual time steps with default settings, to create a baseline. In the AD damage condition, the ADD algorithm was turned on from the beginning and kept on, to simulate the AD condition without intervention. The remaining six conditions corresponded to the six virtual tDCS interventions documented in Figure 2. In each intervention condition, the virtual tDCS was turned on at virtual time point 10, after the ADD algorithm had had time to damage the network (Luppi et al., 2023). The virtual tDCS was done by changing the threshold potential of the excitatory pyramidal neurons (Vd1) of affected neural masses, as determined by the CFM results. Anodally stimulated neural masses had their Vd1 reduced from seven to five (increased excitability), while cathodally stimulated neural masses had their Vd1 increased from seven to nine. The range of these changes was based on our previous modeling work, where we found these changes to result in observable but still physiologically plausible effects (Luppi et al., 2023). The virtual tDCS was kept on for the rest on the simulation. Due to stochastic noise in the model, each stimulation step was repeated 20 times and then averaged for increased reliability and smoother visualization of the results.

### Outcome measures

As in our previous study, the same six outcome measures were used to assess spectral power [total power, relative power in the lower (8–10 Hz) and upper (10–13) alpha bands and posterior dominant peak frequency] and functional connectivity [phase lag index (PLI; Stam et al., 2007) and AEC in the lower alpha band, 8–10 Hz (Bruns et al., 2000; Schoonhoven et al., 2022)]. Uncorrected AEC was used, as there is no volume conduction in the model. These measures were compared between the HC and the ADD with and without intervention conditions in the personalized models. Rather than focusing on absolute values, which can be prone to overfitting and overinterpretation, we primarily used the model to assess directions of change in response to AD damage and virtual tDCS, which it is well suited for. An intervention was considered successful if it resulted in a shift toward the HC values in comparison with the ADD values without intervention. For example, since the ADD algorithm results in a decrease in total power in comparison with the HC condition, a successful intervention would have to cause an increase in total power compared with the ADD condition.

Oscillatory slowing, observed in a shift from higher frequencies (alpha) to lower (delta, theta) especially in occipital regions, is well documented in AD (Jeong, 2004; Babiloni et al., 2013; Engels et al., 2017; Giustiniani et al., 2023) and has been associated with disease severity and pathogenic protein load (Rudolf et al., 2022; Scheijbeler et al., 2023). As the model we use features primarily alpha frequency activity, we have focused on this frequency band, but we also included total spectral power and posterior dominant frequency to assess overall spectral changes. A loss of functional connectivity has also been reported in AD cohorts, including in the measures of our choice, and its extent has been associated with disease severity (Engels et al., 2017; Babiloni et al., 2020; Briels et al., 2020; Schoonhoven et al., 2022). As such, based on the current literature, we argue that being able to elicit a shift in these disrupted outcome measures toward healthy values would also indicate potential for clinical improvement in cognition.

### Quantification of tDCS strategy success

To quantify the relationship between the degree of anodally stimulated regions and stimulation success, each personalized strategy outcome across individuals was given a measure of intervention success in the form of an area under the curve (AUC). We chose to focus on the anodally stimulated regions, because anodal and cathodal stimulation have opposing effects, and in our previous study, anodal stimulation was related to positive stimulation outcomes (Luppi et al., 2023). The change in all outcome measures in response to stimulation was calculated between virtual time points *t* = 10 and *t* = 20, as *t* = 10 is the stimulation onset and the largest stimulation effects are seen between this and *t* = 20. We calculated the AUC of the personalized and general strategy outcome and subtracted the AUC of the ADD outcome from this, to estimate a treatment effect. This was then repeated and averaged for all six outcome measures of each strategy, all six strategies in an individual, and all 10 individuals. This change in AUC was normalized as a *Z*-score to keep the influence of all outcome measures equal. Finally, the average AEC degree of each region that was anodally stimulated in at least one strategy was calculated for the virtual time point *t* = 10 in the ADD without intervention condition. This average AEC degree was then correlated with the averaged AUC *Z*-score of all strategies it was anodally stimulated in. The AUC was chosen as a measure to better capture changes over time from stimulation onset, instead of focusing on a single time point.

### Hub restoration index

To assess whether virtual tDCS was able to restore connectivity to the highly connected and vulnerable hub regions in the model, we adapted a variation of the previously described “hub disruption index” (HDI; Achard et al., 2012; van Nifterick et al., 2024). This measure is calculated as a simple linear regression of the baseline connectivity degree of regions in a control group with the difference in degree of the same regions in a disease group. In contrast, we reversed this measure to consider the AD damage condition without intervention as the control group and the personalized intervention condition as the comparison group. As this comparison measures the effects caused by the intervention, we labeled it the “hub restoration index” (HRI), in which a higher value indicates a better treatment outcome. This index was averaged across all strategies and individuals at virtual time *t* = 15.

### Statistical testing

Due to the model only including stochastic noise as a difference between each run of the simulations, the statistical analysis was kept simple. As mentioned, averaged measures from 20 repetitions were used as basis for all outcome measures, and these effects were further assessed in the form of the AUC changes in each condition and outcome measure. Differences between the AUC outcomes in the ADD without intervention condition, general strategy condition, and personalized strategy condition were then compared using within-subject *t* tests. A Pearson's correlation was calculated between connectivity of anodally stimulated regions and stimulation success. A within-subject *t* test was used to compare the average connectivity of stimulated regions prior to stimulation for the general and personalized strategies. A simple linear regression was calculated for the HRI. Table 1 is a statistical table, summarizing the data structure, statistical test, *p* values, and 95% confidence intervals for all statistical tests in the study,

**Table 1.** Statistical table

Figure/table	Data structure	Type of test	*p* values	Power (95% C.I. of diff)
Figure 4*A*	Normal distribution	Within-subject *t* test	*p* = 0.1920	−0.02294 to 0.09893
Figure 4*B*	Normal distribution	Within-subject *t* test	***p* < 0.0001**	−4.145 to −3.505
Figure 4*C*	Normal distribution	Within-subject *t* test	***p* < 0.0001**	2.690 to –3.037
Figure 4*D*	Normal distribution	Within-subject *t* test	***p* = 0.0001**	58,312 to –122,986
Figure 4*E*	Normal distribution	Within-subject *t* test	***p* = 0.0088**	0.01268 to 0.06646
Figure 4*F*	Normal distribution	Within-subject *t* test	*p* = 0.5617	−0.03677 to 0.06347
Figure 5*A*	Normal distribution	Within-subject *t* test	***p* = 0.0057**	0.09669 to 0.4219
Figure 5*B*	Normal distribution	Within-subject *t* test	***p* < 0.0001**	−4.262 to −3.713
Figure 5*C*	Normal distribution	Within-subject *t* test	*p* = 0.0556	−0.01316 to 0.8951
Figure 5*D*	Normal distribution	Within-subject *t* test	***p* < 0.0001**	208,191 to 424,371
Figure 5*E*	Normal distribution	Within-subject *t* test	***p* = 0.0010**	0.04685 to 0.1307
Figure 5*F*	Normal distribution	Within-subject *t* test	***p* = 0.0049**	0.1776 to 0.7369
Figure 6*A*	Normal distribution	Within-subject *t* test	***p* = 0.0093**	0.06936 to 0.3732
Figure 6*B*	Normal distribution	Within-subject *t* test	***p* = 0.0040**	−0.2581 to −0.06666
Figure 6*C*	Normal distribution	Within-subject *t* test	***p* < 0.0001**	−3.010 to −1.835
Figure 6*D*	Normal distribution	Within-subject *t* test	***p* = 0.0010**	118,937 to 332,326
Figure 6*E*	Normal distribution	Within-subject *t* test	*p* = 0.0547	−0.001237 to 0.09967
Figure 6*F*	Normal distribution	Within-subject *t* test	***p* = 0.0048**	0.1733 to 0.7144
Figure 10	Normal distribution	Pearson’s correlation	***p* = 0.0004**	0.2066 to 0.6169
Table 3	Normal distribution	Within-subject *t* test	***p* < 0.0001**	−0.03499 to −0.02299
Figure 11	Normal distribution	Simple linear regression	***p* < 0.0001**	0.6180 to 0.9492

This table displays the following information for all statistical tests performed: data structure, type of test used, *p* values, and the power in the form of 95% confidence intervals of the difference in means. Significant *p* values are shown in bold.

### Code accessibility

As the data were generated using the graphical interface of the BrainWave software, there is no relevant code to be made available. However, the Brainwave software is made freely available at https://github.com/CornelisStam/BrainWave. The generated data have also been made available on a Zenodo repository at https://doi.org/10.5281/zenodo.14861689.

## Results

We first provide an overview of the averaged simulation outcomes in the new personalized models. We compare the best personalized strategy in each model with the previously established general strategy in the same models. Then, we further explain these results in the context of the underlying connectomes used in the new personalized and the old general models.

### Personalization leads to different tDCS predictions and performance

The personalized strategy of each model is the best-performing strategy of the said model, i.e., the strategy that resulted in most improvement toward HC values across all outcome measures. In eight cases, this was F7 anode with F4 cathode; in one case, it was F8 anode with F3 cathode; and in one case, it was PO8 anode with AF3 cathode, the same as the general strategy.

**Figure 3.**
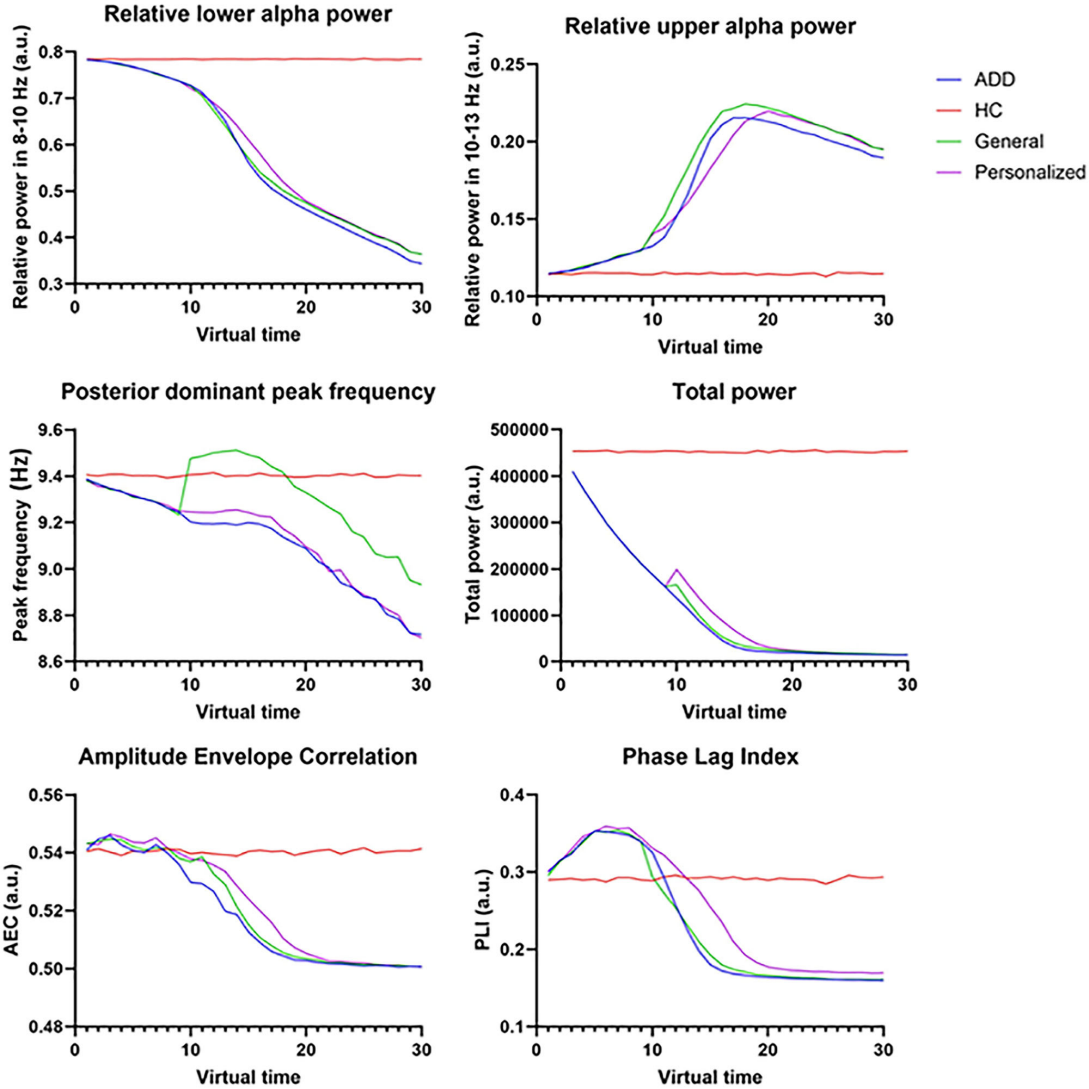
Averaged outcome measures between all individuals for personalized and general strategies. Stimulation onset was at virtual time point 10. The best-performing personalized strategy and the general strategy imported into the personalized model are shown. A strategy was successful if it caused a shift from the values of the ADD condition without intervention and toward the HC condition. AEC, amplitude envelope correlation; ADD, activity-dependent degeneration; HC, healthy control.

Figure 3 displays the results for all six outcome measures, averaged across the 10 personalized models. The personalized strategies resulted in a shift away from the AD damaged conditions and toward HC values in all outcome measures, therefore showing overall improvement. This change was in general present from stimulation onset at virtual time point *t* = 10 to approximately *t* = 20, at which point the ADD algorithm overpowered the effects of the intervention due to the lack of structural plasticity in the model. The personalized strategies outperformed the general strategies in relative lower and upper alpha power, total power, AEC, and PLI, with the exception of the posterior dominant peak frequency, where the opposite was true. As such, personalizing the model resulted in (1) different preferred stimulation strategies and (2) better-performing strategies, although the differences were not large in the harsh ADD damage regime.

**Figure 4.**
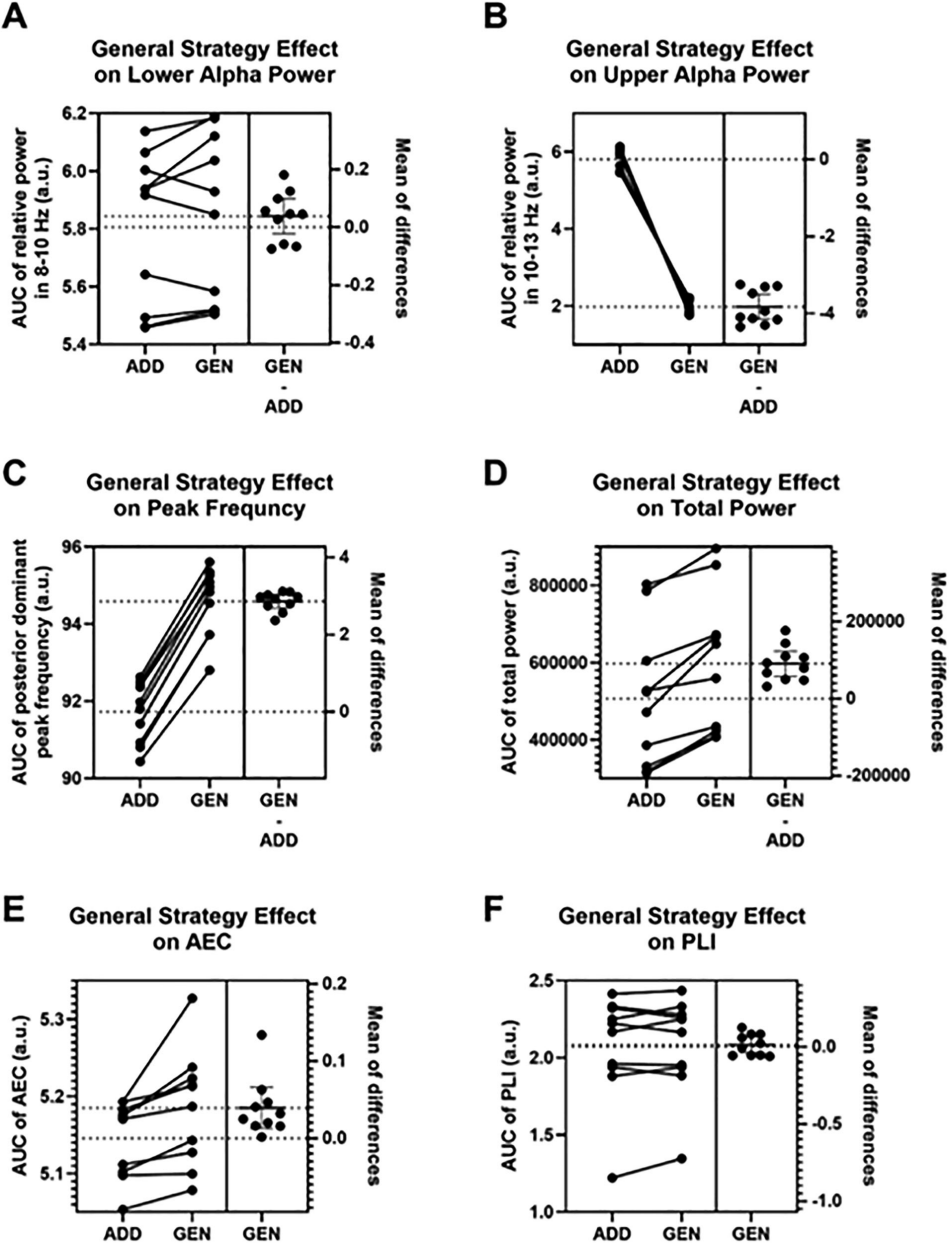
Estimation plot of the AUC differences between general strategy and no intervention conditions. AUC values were calculated between time pints 10 and 20 for relative power in the (***A***) lower alpha band and (***B***) upper alpha band, (***C***) posterior dominant peak frequency, (***D***) total power, (***E***) AEC, and (***F***) PLI. a.u., arbitrary units; ADD, AD damage without intervention; GEN, general strategy.

**Figure 5.**
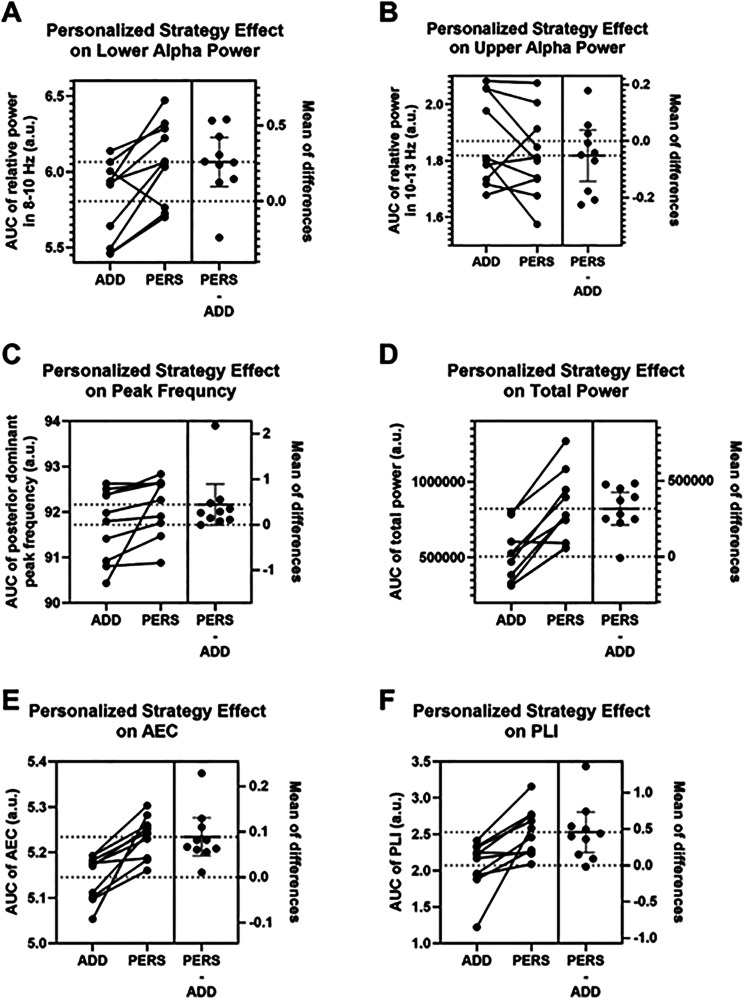
Estimation plot of the AUC differences between personal strategy and no intervention conditions. AUC values were calculated between time pints 10 and 20 for relative power in the (***A***) lower alpha band and (***B***) upper alpha band, (***C***) posterior dominant peak frequency, (***D***) total power, (***E***) AEC, and (***F***) PLI. a.u., arbitrary units; ADD, AD damage without intervention; PERS, personalized strategy.

**Figure 6.**
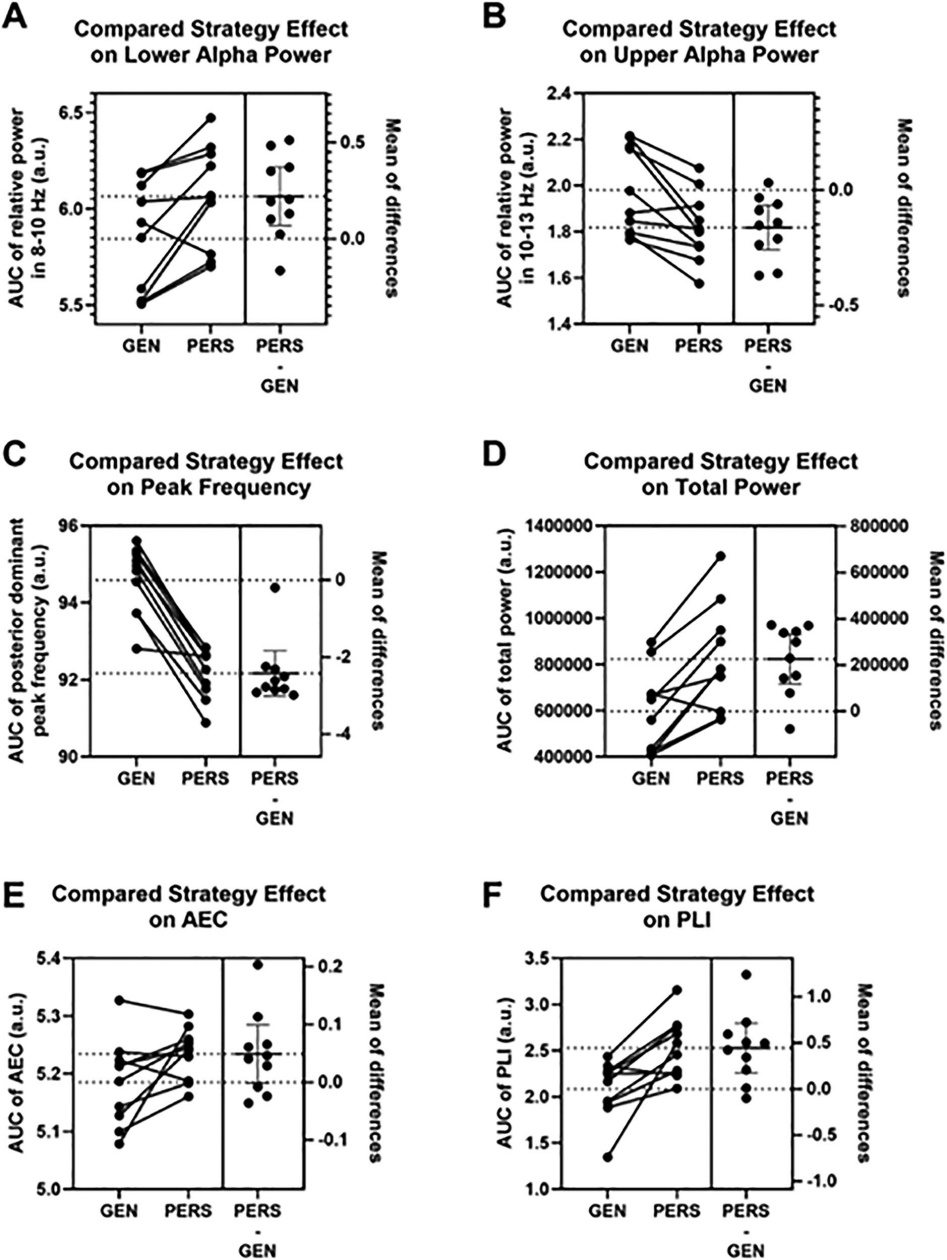
Estimation plot of the AUC differences between personal and general strategy conditions. AUC values were calculated between time pints 10 and 20 for relative power in the (***A***) lower alpha band and (***B***) upper alpha band, (***C***) posterior dominant peak frequency, (***D***) total power, (***E***) AEC, and (***F***) PLI. a.u., arbitrary units; GEN, general strategy; PERS, personalized strategy.

To assess the effect sizes of the general and personalized strategies in response to the no intervention condition and each other, we calculated the AUC between time points 10 and 20 for all outcome measures. These comparisons are shown in Figures 4–6, and the relevant values along with the *p* values for within-subject *t* tests are summarized in Table 2. The general strategy resulted in significant improvements in peak frequency, total power and AEC, while a significant shift further away from the HC values was seen in relative power in the upper alpha band. The personalized strategy resulted in significant improvements in all outcome measures, except for peak frequency, where the improvement was not significant. The personalized strategy significantly outperformed the general strategy, except for the peak frequency, which was more improved in the general strategy, and the PLI, where the difference was not significant.

**Table 2.** Effect sizes of tDCS strategies

	Relative low alpha power (8–10 Hz)	Relative high alpha power (10–13 Hz)	Posterior dominant peak frequency	Total power	AEC	PLI
ADD without intervention AUC	5,8 (±0,26)	1,9 (±0,16)	92 (±0,79)	51 × 10^4^ (±18 × 10^4^)	5,14 (±0,05)	2,07 (±0,35)
General strategy AUC	5,84 (±0,29)	1,98 (±0,19)	95 (±0,89)	60 × 10^4^ (±18 × 10^4^)	5,19 (±0,07)	2,08 (±0,32)
Personal strategy AUC	6,06 (±0,27)	1,82 (±0,15)	92,17 (±0,64)	82 × 10^4^ (±23 × 10^4^)	5,23 (±0,05)	2,53 (±0,33)
General versus ADD	+0,7% *p* = 0.1920	−5,9% *p < 0.0001	+3,1% *p < 0.0001	+17,9% *p = 0.0001	+0,8% *p = 0.0088	+0,6% *p* = 0.5617
Personal versus ADD	+4,5% *p = 0.0057	+2,8% *p < 0.0001	+0,5% *p* = 0.0556	+62,4% *p < 0.0001	+1,7% *p = 0.0010	+22,1% *p = 0.0049
Personal versus general	+3.8% *p = 0.0093	+8.2% *p = 0.0040	−2.6% *p < 0.0001	+37.8% *p < 0.0010	+0.9% *p* = 0.0547	+21.3% *p = 0.0048

The AUC values were averaged across all subjects for three conditions: the AD damage (ADD) condition without intervention, the general intervention strategy, and the individually best-performing personalized strategy. These are listed in the first three rows, along with their standard deviations. The AUC was calculated between timepoints *t* = 10 (stimulation onset) and *t* = 20. In the last three rows, the percentage change from the ADD mean is shown for both intervention conditions, as well as for the difference between intervention conditions. A positive value denotes improvement (an increase, except for high alpha, for which the value was made positive to fit the others). The *p* value for a within-subject *t* test is shown for all contrasts. *, a significant change.

**Figure 7.**
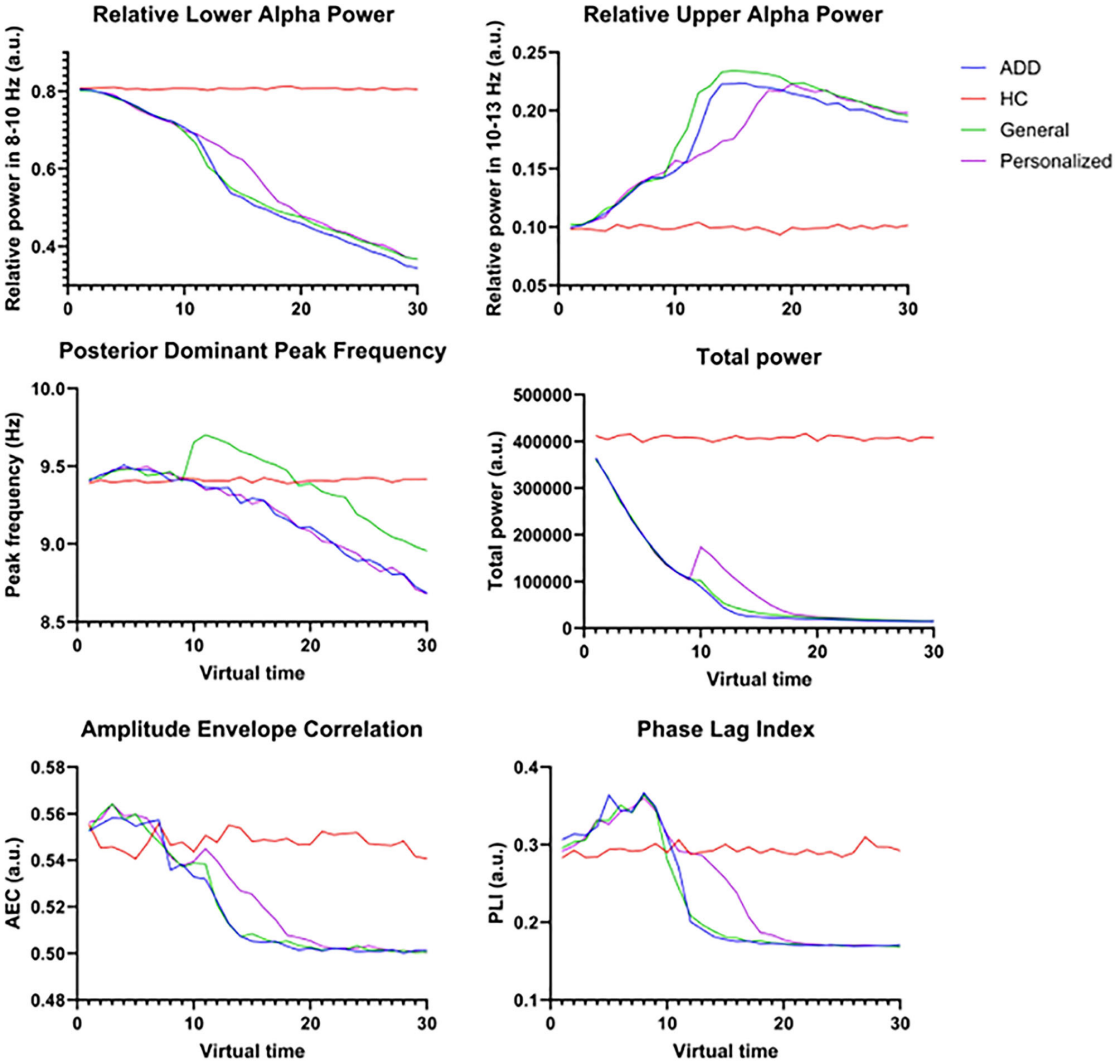
Outcome measures of a representative individual case for personalized and general strategies. Stimulation onset was at virtual time point 10. The best-performing personalized strategy and the general strategy imported into the personalized model are shown. A strategy was successful if it caused a shift from the values of the ADD condition without intervention and toward the HC condition. AEC, amplitude envelope correlation; ADD, activity-dependent degeneration; HC, healthy control.

To demonstrate that the results were not highly variable between subjects, we have included an example of a representative individual case. This is Figure 7, which was otherwise identical to Figure 3, except for being based on a single subject, instead of averaged results.

### Connectome source (DTI vs MEG) choice influences tDCS performance

**Figure 8.**
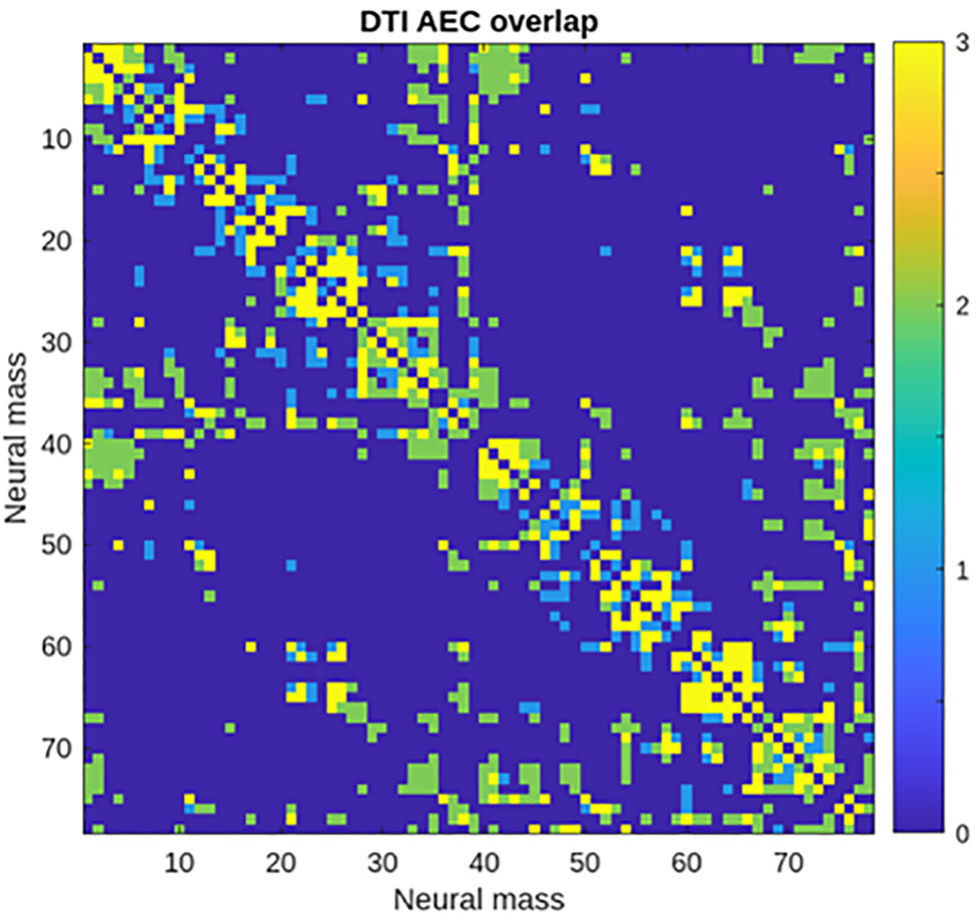
Overlap between the original DTI-based connectivity matrix and averaged personalized AEC-based connectivity matrix. A dark blue square denotes a lack of connection in both matrices, a light blue point denotes a connection only in the DTI matrix, and a green square denotes a connection only in the AEC matrix, while a yellow point denotes a connection shared by both matrices.

Due to the difference in best-performing stimulation strategies in the personalized and the general models, the differences in their connectivity were investigated. Figure 5 displays the overlap between the connectivity matrices based on the general DTI and the average of the personalized AEC results. While the matrices have a similar overall structure, one key difference is that the AEC matrix features more strong frontal connections (please see the green frontal clusters in Fig. 8).

### Comparison with a model with an averaged AEC matrix

**Figure 9.**
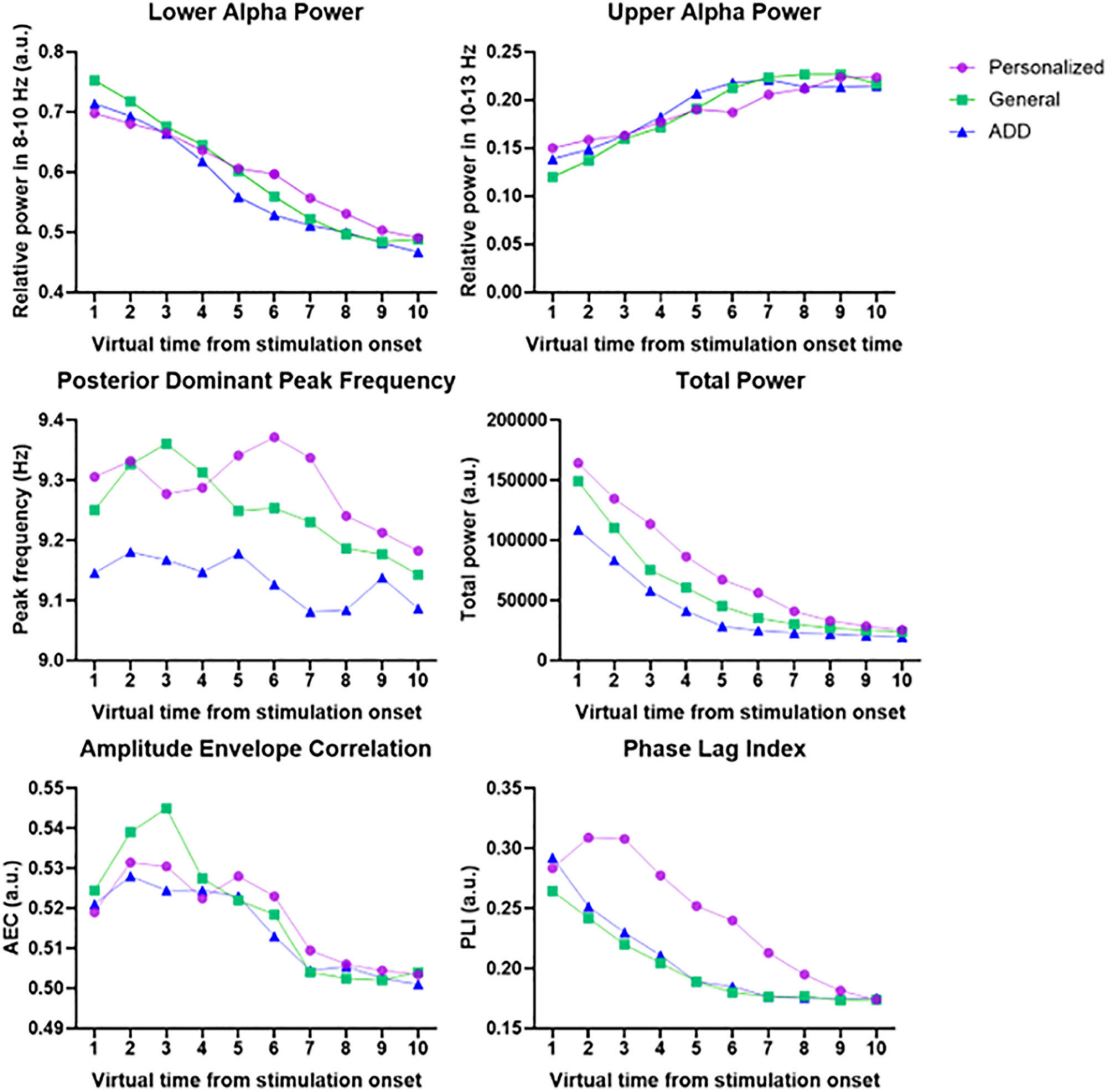
Outcome measures using an averaged AEC matrix from 10 subjects. The best-performing personalized strategy (F7 anode with F4 cathode, which also performed best in this model) and the general strategy imported into the personalized model (PO8 anode with AF3c cathode) are shown over the first 10 virtual time epochs after stimulation onset. AEC, amplitude envelope correlation; ADD, activity-dependent degeneration.

Figure 9 displays results in all outcome measures over 10 epochs from the stimulation onset in an alternative model in which an averaged matrix calculated from the AEC of all 10 subjects was used. These figures were generated for 10 virtual time points from the stimulation onset (time point 10 in Fig. 3). While there are many similarities to Figure 3, for example, in that the F7 anode with F4 cathode montage performed best and the relationships between conditions were identical for total power, differences are also present. Unlike in Figure 3, posterior dominant peak frequency does not favor the personalized strategy as clearly, and AEC favors the general strategy.

### Network topography influences tDCS performance

**Figure 10.**
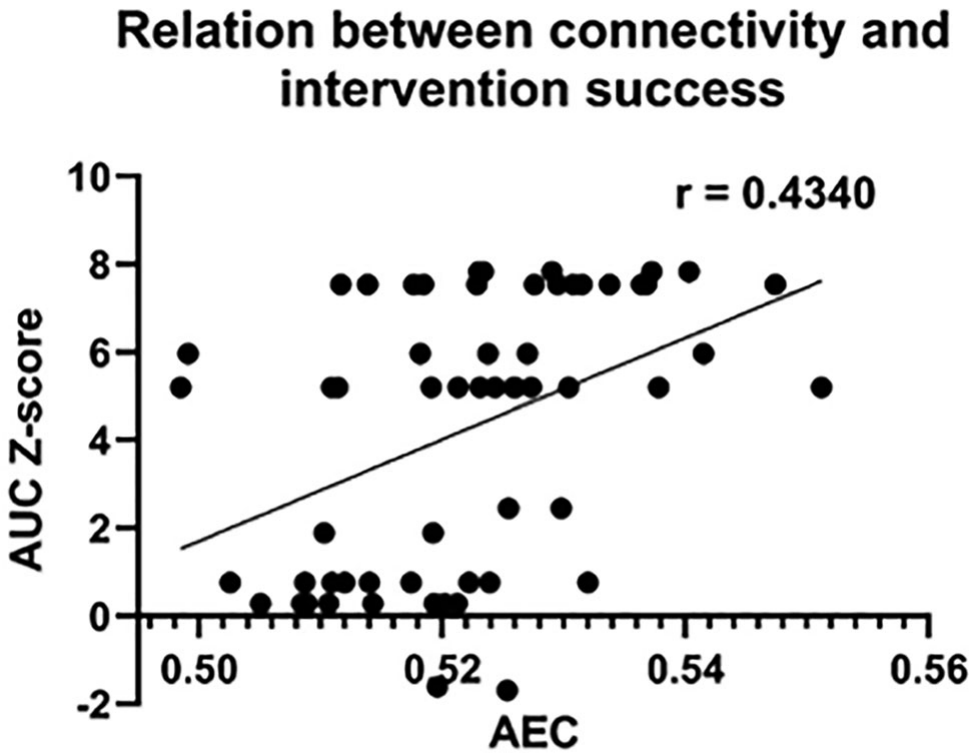
Correlation between the AEC degree of anodally stimulated regions and the AUC *Z*-score as a measure of personalized intervention success. Each personalized strategy was given a measure of intervention success in the form of an averaged AUC change across all outcome measures in those strategies between virtual time points *t* = 10 and *t* = 20, which was normalized as a *Z*-score. The average AEC degree of each region that was anodally stimulated in one or more of the personalized strategies was correlated with the average AUC *Z*-score in those strategies. A significant, moderately strong positive Pearson’s correlation was observed (*r* = 0.434; *p* < 0.001).

Next, we quantified the relationship between stimulation success and the averaged degree centrality (AEC, 8–10 Hz) of anodally stimulated regions, in the form of an averaged AUC change. As seen in Figure 10, significant, moderately strong positive Pearson's correlation was observed (*r* = 0.434; *p* < 0.001), indicating that anodally stimulating higher degree regions resulted in a greater intervention success.

Table 3 shows the ranking of anodally stimulated regions by degree for both the personalized strategy (F7 anode, F4 cathode) and the general strategy (PO8 anode, AF3 cathode), in a representative individual case. The average AEC degree prior to stimulation was significantly higher in the areas that were anodally stimulated in the personalized strategy (within-subject *t* test, *p* < 0.0001).

**Table 3.** Ranking of the anodally stimulated regions by degree

Anodally stimulated neural masses in personalized strategy	Associated AEC degree	Anodally stimulated neural masses in general strategy	Associated AEC degree	*p* value
Middle temporal pole gyrus L	0.581	Rolandic operculum R	0.585	
Superior temporal pole gyrus L	0.577	Heschl gyrus R	0.568	
Rolandic operculum L	0.574	Superior temporal gyrus R	0.539	
Heschl gyrus L	0.569	Middle temporal gyrus R	0.536	
Rectus L	0.569	Postcentral gyrus R	0.530	
Temporal superior gyrus L	0.562	Superior occipital gyrus R	0.527	
Frontal medial orbital gyrus L	0.561	Supramarginal gyrus R	0.524	
Frontal inferior orbital gyrus L	0.559	Lingual gyrus R	0.524	
Frontal inferior triangulate gyrus L	0.559	Cuneus R	0.521	
Frontal superior orbital gyrus L	0.556	Precuneus R	0.519	
Frontal middle orbital gyrus L	0.545	Inferior parietal gyrus R	0.518	
Frontal superior medial gyrus L	0.541	Parietal superior gyrus R	0.514	
Postcentral gyrus L	0.540	Angular gyrus R	0.511	
Inferior temporal gyrus L	0.539	Calcarine fissure R	0.511	
Insula L	0.539	Middle occipital gyrus R	0.508	
Frontal superior gyrus L	0.529	Inferior occipital gyrus R	0.504	
Mean	0.556	Mean	0.527	<0.0001

For a representative individual case, the regions that were anodally stimulated in the personalized strategy (F7 anode, F4 cathode) and the general strategy (PO8 anode, AF3 cathode) were ranked by degree as measure by AEC. Degree was measured at virtual time *t* = 9, prior to stimulation onset. L, left hemisphere; R, right hemisphere. The mean degree was significantly higher for the regions anodally stimulated in the personalized strategy (within-subject *t* test, *p* < 0.0001).

**Figure 11.**
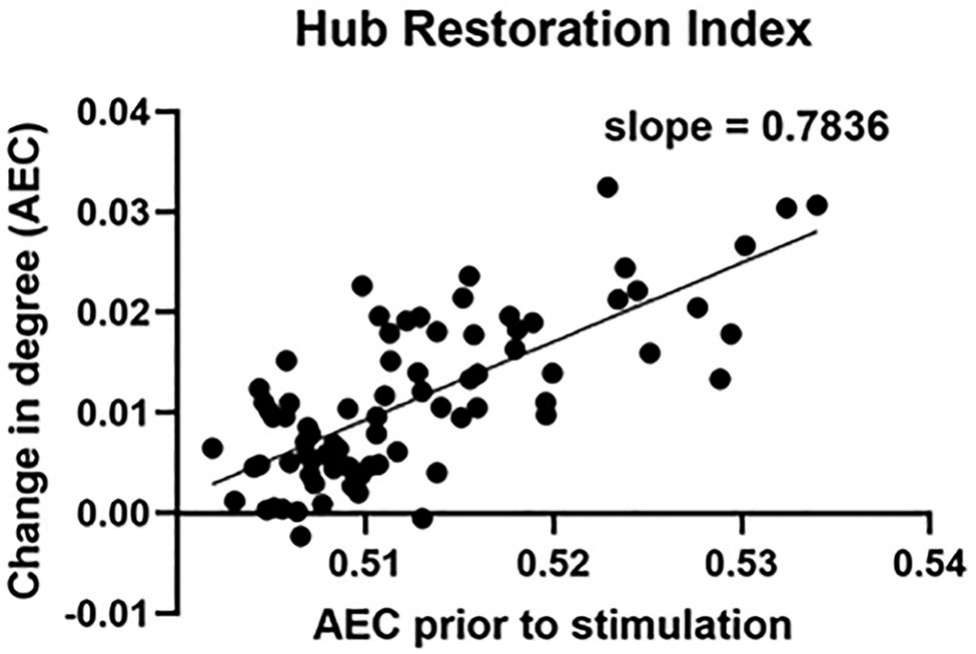
HRI for the anodally stimulated regions. To assess the topological consequences of stimulation, an HRI was calculated. For each region, its AEC degree in the control condition was correlated with the comparative average change in AEC degree in all personalized interventions it was anodally stimulated (both taken at virtual time point *t* = 15). A significant slope of 0.7836 (*p* < 0.0001) was found, indicating that anodally stimulating high degree regions resulted in a stronger restoration of AEC connectivity.

Figure 11 displays the HRI, which had a strong, significant slope of 0.7836 (*p* < 0.0001), indicating that the hub regions with a higher AEC degree without intervention responded to anodal stimulation with the highest increase in AEC degree.

## Discussion

In this study we aimed to explore the influence of implementing individual brain structure and connectivity in a previously published computational model simulating tDCS treatment in AD, to improve the prediction of tDCS treatment response. The stimulation effects of the personalized strategies resulted in improvement in all outcome measures compared with the “no intervention” condition and in all outcome measures except the posterior dominant peak frequency compared with the general strategy condition. Personalizing the model resulted in a dorsolateral prefrontal cortex (dlPFC)-targeting electrode setup performing best in all but one individual (for whom the general strategy performed best), contrasting the results of the general model, which favored a precuneus-targeting montage. Favorable treatment response was correlated with anodal stimulation of highly connected hub regions and appeared to be especially restorative for the functional connectivity of these same regions. Here, we discuss the implications of our findings, their relation to existing literature, limitations, and potential future directions.

### Model personalization leads to different tDCS strategies

We found that personalizing the neural mass model based on individual AEC-based network connectivity resulted in a different strategy performing best in nine out of ten individuals, compared with our previous results in the nonpersonalized model. Instead of the previously most successful general strategy [with a posterior anode near the precuneus (PO8) and a contralateral frontal cathode (AF3)], a strategy with a temporofrontal anode (F7 or F8) and a frontal cathode (F4 or F3) performed best. These frontal strategies involve the dlPFC, which is one of the popular targets for tDCS interventions due to its involvement in the default mode network (Javadi and Walsh, 2012; Murugaraja et al., 2017; To et al., 2018; Im et al., 2019). These strategies were also successful in the general model but were outperformed by the precuneus-targeting montages in effect size (Luppi et al., 2023). Furthermore, targeting the dlPFC has been effective in tDCS treatments of depression (Zheng et al., 2024). Overall, these results indicate that introducing personal network connectivity notably affects the predictions, but the consistency within the personalized group might also imply that the primary cause of the observed difference is the choice for functional data instead of structural and not the individual connectivity details (Antonenko et al., 2021; Antal et al., 2022).

When comparing the connectivity matrices of the personalized and the general models, certain features stood out as differences. The overall structure of the matrices was quite similar, as can be appreciated in Figure 8. However, the sparsity of the matrices was 10% for the general and 15% for the personalized matrices. This was done to match the average connectivity degree between the two, since measures can be dependent on network size and density. The general matrix was binarized between values of 0 and 1, while the personalized matrix values were kept continuous in order to retain information, with average values for active connections being closer to 0.5 than 1. Therefore, more connections needed to be included in the personalized matrix to balance network density by adjusting the threshold to include the strongest 15% of the connections, but this may have had other unanticipated effects on the results. Moreover, the personalized matrices included topological differences, such as more frontal connectivity clusters than the general matrix. By investigating the connections with the highest degree, it was also confirmed that the connections present in the personalized but not the general matrix were not simply weaker connections that were introduced as the matrix was made less sparse. This presence of higher frontal connection density is relevant, as it could explain why frontal stimulation strategies performed better in the personalized model. While the connectivity matrices might also be different because they are derived from different modalities, we decided to accept this difference, because ultimately, we are interested in finding out whether adding different types of empirical data (i.e., MRI, MEG) helps restoring functional network integrity, regardless of dissimilarities. Of course, whether the observed difference between these strategies will be similar and equally effective in the clinical experiments is yet unknown, but from the future discrepancies between the virtual and clinical results, we hope to understand which methodological choices are most valid.

### Connectivity matrix differences: driven by population or methodology?

There are three key differences in how the connectivity matrices of the personalized and general models were made: the personalization itself, as well as the population and the connectivity measure and modality the matrices are based on. Firstly, the personalized model aims to be more suited to the individual and therefore could lead to different and potentially more accurate predictions of intervention outcome. This perspective is difficult to validate before the upcoming clinical trial comparing the efficacy of the personalized and general models in predicting optimal stimulation strategies. However, the personalization could already have an effect on the results through differences in the population used for the matrices. The general human DTI matrix was based on data form healthy adults, whereas the personalized AEC matrices are based on 55–80 old patients with a biomarker-confirmed AD diagnosis. Therefore, the personalization process could be integrating features of AD pathology and aging that were not present in the general matrix. Interestingly, some studies report increases in frontal connectivity that are theorized to be a form of compensation and predict less severe AD progression (Franzmeier et al., 2018), while others claim that such increases predict pathology (Mukli et al., 2024). Therefore, incorporating such features into the model via personalization could benefit both prediction accuracy and our understanding of regional connectivity changes in AD.

Another possible explanation for the observed differences could be caused by the different connectivity measures used. In the general model, a purely structural connectivity measure was used, while the personalized model uses uncorrected AEC as a proxy for structural connectivity. This is because while a corrected AEC is a functional connectivity measure, leaving out the volume conduction correction results in a matrix more closely related to structural connectivity. Naturally, this can result in differences between the MRI-based DTI and the MEG-based AEC, as well as the remaining influence of functional connectivity on AEC. Moreover, uncorrected AEC is predisposed to finding spurious, false-positive connections in the midline of the brain. The reliability for the MEG beamformer reconstruction of deep sources is smaller, especially near the midline, and nearby virtual channels look more similar than they should. This can lead to spurious connections when using uncorrected FC measures and could be behind at least some of the frontal connections that were observed in the AEC matrices but not in the DTI matrix.

To address this question, we additionally ran simulations in a model based on an averaged AEC matrix across our study population, as shown in Figure 9. This served as a comparison to see whether personalized models would still have different results when compared with an averaged connectivity model based on the same AEC methodology. As could be expected, results from this model were similar to the results of the personalized models (Fig. 3), but differences were also present. The best-performing strategy, as in 90% of the personalized models, was the F7 anode F4 cathode strategy, and many of the relationships between the conditions were the same. For example, total power was highest in the personalized strategy, second-highest in the general strategy, and lowest in the condition without intervention. However, while posterior dominant peak frequency was highest in Figure 3 in the general strategy, in the new averaged AEC model, it was highest in the personalized strategy. Additionally, AEC results in Figure 3 were highest in the personalized strategy, while in the average AEC model, they were highest in the general strategy. As such, while these results are not conclusive and would benefit from a larger sample size, the differences observed indicate that differences between the personalized and general model outcomes are not based solely on the different methodologies used.

### Hub status of stimulated regions correlates with intervention success

A moderately strong significant correlation was found between the degree of anodally stimulated regions and their involvement in successful personalized strategies. In essence, the more highly connected regions were more likely to be anodally stimulated in the best-performing strategies. This was also observed in the finding that the average degree of anodally stimulated regions was higher for the personalized strategy than the general strategy in a representative individual case. Again, this could support the explanation that the higher frontal connectivity in the personalized model compared with the general model resulted in more frontal strategies performing better. It could be that stimulating the less damaged frontal regions is more effective in spreading the beneficial effects of the tDCS intervention throughout the network. This may be even more important in individuals with more advanced AD pathology, as the connectivity of the personalized models was based on AD patients instead of healthy adults. Interestingly, we performed the same comparison in our previous publication on the general model and found no such correlation (Luppi et al., 2023). This remains the case even when repeating our updated methodology of using AUC scores on the previous dataset. It is not clear what is behind this difference, but it could be related to the aforementioned differences in population and connectivity measure. Intuitively, targeting hub regions is in theory a sound strategy to improve controllability due to their high degree of connections with the rest of the target network (Hagmann et al., 2008; van den Heuvel and Sporns, 2011; Gu et al., 2015), and recent studies using tDCS in chronic pain have shown support for this approach (Muldoon et al., 2016; Kaplan et al., 2020). However, we did not define our strategies based on connectivity, and whether this finding is true in the case of the current model and AD requires upcoming clinical validation, especially given the possibility of them being influenced by the aforementioned midline artifacts

### Increase in hub connectivity

Selective hub vulnerability is a well-established phenomenon in AD, making hub regions a potential therapeutic target. Recent studies exploring the role of disrupted hub connectivity have used the HDI as a tool for investigating the selective global and regional vulnerability of highly connected brain network regions in AD (Achard et al., 2012; van Nifterick et al., 2024). This concept is closely tied to the damage caused by the ADD algorithm in our model, as damage based on the activity level affects hubs more severely than less connected regions (de Haan et al., 2012; Engels et al., 2015). To counter this, network control theory supports targeting hubs for maximal network influence (Gu et al., 2015; Betzel and Bassett, 2018). We therefore introduced a reversed version, the HRI, to assess whether our interventions could help improve hub connectivity. We found a significant slope of 0.7836, with anodal stimulation of higher degree regions resulting not only in connectivity improvement but also restoring neuronal dynamics relatively effectively. This lends support to the personalized interventions being able to intervene at the level of a key neurophysiological feature of AD. Naturally, this is only one potential way in which network health can be improved. For example, interest in intervening at the level of excitation–inhibition (E/I) balance, to reduce metabolic demand and optimize neuronal firing response repertoires, has been increasing in previous years, due in part to its potential in attenuating the metabolically high demands of hub hyperexcitability (Van Nifterick et al., 2022; van Nifterick et al., 2023, 2024).

### Limitations and future directions

Naturally, the methodologies used in this experimental new approach to personalizing tDCS in AD are not without limitations. Firstly, although the focus is on individual results, the study could benefit from a higher number of participants included and strategies tested. For this personalization study, we opted for a modest number of strategies and individuals, as increasing their number causes a multiplication of the already considerable number of separate simulations and analyses needed. However, the strategies tested were selected from the 6 out of 20 strategies that performed best in the general model, and during preliminary analyses, these 6 strategies also performed the best in the personalized models. Secondly, the translation of the CFM results to the neural mass model changes involves laborious fitting of the CFM results to the AAL atlas used in the model. Both of the issues could possibly be alleviated if some of the processes could be further automated. For example, instead of working with fixed electrode montages, some CFM software solutions allow for optimizing montages to target specific regions. This approach could help limit the number of montages that need to be simulated and analyzed per participant while simultaneously improving the accuracy of the targeting. This could be further improved by choosing another atlas as a basis for the model, preferably with more uniform regions shape and size than the AAL atlas, leading to an easier and more accurate conversion of the CFM results into model settings.

Furthermore, the difference in study population and connectivity measure of the personalized and general matrices make it challenging to compare and explain the results between the two. This was a necessity caused by a lack of access to DTI data and the need to include AD patients and not healthy adults in the study. This comparison could be made more straightforward by generating a new connectivity matrix based on AEC or another connectivity measure in a larger sample of the AD population and then using this as a basis for the general model. While we addressed this issue with and average AEC matrix based on our study population, these differences must be held in mind when interpreting our results.

Finally, while the assumption that restoring network function will lead to clinical improvement in cognition is logical, it has not yet been proven. Ultimately, our predictions will naturally need to be validated in the upcoming clinical trial comparing MEG measurements during personalized and general tDCS interventions, which can also help inform us about optimal methodology for further personalization of the interventions.

While the BrainWave software used in this study is made available for free use, we acknowledge that going toward a more systematic and unified use of toolboxes for analysis across research groups would be beneficial. For example, noninvasive brain stimulation toolboxes such as BEST (Hassan et al., 2022) and MEG analysis toolboxes such as FLUX (Ferrante et al., 2022) could help in bringing results such as these to other groups in a more standardized form. BrainWave was used due to our groups familiarity with it, but more widely established options hold merit for future works.

Finally, it is good to keep in mind that the present study focuses on short-term neuromodulation effects, while we know that most clinical gain is to be expected with repeated, longer stimulation regimes. Neuroplasticity, which might be one of the main mechanisms to counter neurodegeneration, is not taken into consideration in this work. This is because adding plasticity greatly complicates a model and can easily make the network unstable, although in an ideal case its inclusion would make the model more realistic. As such, instantaneous, direct neuromodulatory effects of tDCS were the focus of this study, as they will be in the clinical validation phase in which we will record the effects of ongoing tDCS on AD patients' brain activity as measured via MEG. We believe that, given the neuronal hyperexcitability in AD, beneficial changes can arise from the neuromodulatory effect of tDCS, which in turn can lead to longer-term changes via plasticity.

### Conclusion

We have described a novel methodological approach for integrating individual brain anatomy and connectivity into a neural mass model-based approach of optimizing tDCS interventions in AD for further personalization. We observed that the personalized approach resulted in a different, more frontal electrode montage being favored over a more posterior montage previously indicated as the best in a general, nonpersonalized model. These differences appear to be related to the connectivity of the anodally stimulated regions, and the outcomes demonstrate potential in restoring connectivity of damaged hubs. General and personalized virtual tDCS model predictions will be validated in an ongoing sham-controlled clinical tDCS–MEG trial, in which MEG recordings during and immediately following general and personalized strategies will be compared.

## Synthesis

Reviewing Editor: Alexander Soutschek, Universitatsklinikum Wurzburg Decisions are customarily a result of the Reviewing Editor and the peer reviewers coming together and discussing their recommendations until a consensus is reached. When revisions are invited, a fact-based synthesis statement explaining their decision and outlining what is needed to prepare a revision will be listed below. The following reviewer(s) agreed to reveal their identity: Ganesan Venkatasubramanian.

Reviewers agreed that the work has the potential to further the field of personalized neuromodulation. However, the lack of detailed results about potential clinical relevance and the lack of a healthy control group to provide a 'normal' baseline for comparison were considered major weaknesses. Moreover, the paper contains several unsupported statements about neuromodulation that need to be clarified and/or supported by literature.
